# Comparison of supraintercondylar and supracondylar femur fractures treated with condylar buttress plates

**DOI:** 10.1186/s12891-016-1278-2

**Published:** 2016-10-04

**Authors:** Chun-Jui Weng, Chi-Chuan Wu, Kuo-Fun Feng, I-Chuan Tseng, Po-Cheng Lee, Yu-Chih Huang

**Affiliations:** Department of Orthopedic Surgery, Chang Gung Memorial Hospital, Chang Gung University, No. 5, Fu-Hsin Street, Kweishan, Taoyuan Taiwan

**Keywords:** Supraintercondylar fractures, Supracondylar fractures, Femur, Condylar buttress plates

## Abstract

**Background:**

Treatment of supraintercondylar (AO/OTA 33-C) and supracondylar (AO/OTA 33-A) femur fractures is generally challenging. Standard treatments include open reduction and internal fixation. However, optimal implants are now being well-defined. This study focus on the comparison between clinical and functional outcomes of fractures treated with condylar buttress plates (CBPs).

**Methods:**

We treated 87 patients with supraintercondylar or supracondylar femur fracture from 2004 to 2008, including 30 supraintercondylar and 24 supracondylar fractures treated with CBPs. Both knee and function scores (per Knee Society) were given to clinical and functional outcomes, and concomitant knee function was assessed per Mize criteria.

**Results:**

Union rate of supraintercondylar fractures was 90 % (27/30) and supracondylar fractures was 91.7 % (22/24) (*P* = 0.68). In supraintercondylar group, 16.7 % revealed postoperative varus deformity, whereas none in supracondylar group (*P* = 0.045). Knee Society knee score was 73.6 in supraintercondylar group and 85.5 in supracondylar group (*P* = 0.009); and function score was 62.5 in supraintercondylar group and 83.1 in supracondylar group (*P* = 0.023). A satisfactory result based on modified Mize criteria was achieved in 50 % of supraintercondylar fractures and in 79.1 % of supracondylar fractures (*P* = 0.09).

**Conclusions:**

Use of CBPs for supraintercondylar and supracondylar femur fractures treatment led to a high union rate. However, a high rate of varus deformity occurred in patients with supraintercondylar but not supracondylar fractures. Moreover, CBP treatment in patients with supracondylar fractures led to better functional outcomes than those with supraintercondylar fractures.

## Background

Treatment of distal femur fractures remains clinically challenging. Owing to improvements in surgical techniques and modern implant designs, open reduction and internal fixation (ORIF) is thought to be the standard treatment by many orthopedic surgeons. In 1960s, in a series of 213 cases, Stewart and colleagues compared surgical and conservative treatment of distal femur fractures and concluded that Kirschner pin traction was recommended as the treatment of choice, with higher acceptable results than with ORIF [1]. Neer and colleagues also reached the same conclusion [2]. They analyzed 110 cases and found that compared with operative treatment, non-operative treatment led to superior outcomes (54 vs. 90 %, respectively). However, surgical techniques and implants were not as good as modern days.

In 1970s, Arbeitsgemeinschaft für Osteosynthesefragen (AO) reported good or excellent results in 74 % of 112 supracondylar femur fractures treated with a condylar plate. Studies by Schatzker and colleagues [3] reported good or excellent results in 73.5–75 % patients following ORIF. He emphasized the importance of early motion and stable fixation. Since the 1970s, ORIF has gained increasing popularity. Various types of internal fixations have been used to achieve anatomic reduction and rigid fixation. Plate systems are the favored method of treatment, including condylar buttress plates (CBPs), dynamic condylar screws, fix-angle condylar plates, and locking plates [3–12].

According to our previous study, AO/OTA 33-C fracture, also called supraintercondylar femur fractures, treated with a CBP resulted in a union rate of 90 % [12]. However, a high incidence of varus deformity (16.7 %) also was noted. A review of the current literature revealed no published studies that compare clinical and functional outcomes between AO/OTA 33- C(supraintercondylar fractures) and AO/OTA 33-A fracture (supracondylar femur fractures) treated with a CBP. The aim of the present study was to retrospectively evaluate clinical and functional outcomes of both types of fracture treated with a CBP and determine the suitability of such a treatment modality.

## Methods

During 2004/3-2008/11, we treated 87 consecutive adult patients (>16 years of age) with closed supraintercondylar or supracondylar femur fracture at our institution. Among these, 60 were treated with ORIF with a CBP. The remaining 27 patients were treated with another internal fixator, such as a locking plate, fixed-angled plate, dynamic condylar plate, or retrograde nailing. We excluded those who were lost to follow up with a year of the surgery. Finally, 54 patients were included in the final evaluation. Among these, 30 had supraintercondylar and 24 had supracondylar fractures. All fractures were categorized according to the AO classification [13].

In the emergency department, the vital signs of each patient were stabilized first. A lower leg skin traction or long leg splint was applied after admission. Operative treatment was arranged for as early as possible. All operations were performed by a group of experienced surgeons with a traditional lateral approach. The patient was placed in the supine position and a pneumatic tourniquet was used. A CBP (Synthes, Bettlach, Switzerland) was applied after fracture reduction. Supplementary screw fixation was used for additional support in complicated fractures. Postoperatively, knee immobilization was not needed.

Active range of motion exercise was initiated after surgery and patients were encouraged to ambulate with a walker or crutches. Radiographs were taken postoperatively during hospitalization and at every outpatient department (OPD) visit. The first OPD visit was arranged within 2 weeks after discharge from the hospital, and the interval between every OPD visit was prolonged until bony union was achieved.

Fracture union and bony alignment were evaluated radiographically by performing anteroposterior and lateral plain radiographs of the injured leg. We defined fracture union as trabeculae crossing the fracture site on serial roentgenograms. On the contrary, fracture nonunion was defined as the absence of trabeculae crossing the fracture on plain radiographs at postoperative 1-year or as the need for revision surgery within 1 year after primary ORIF surgery. With respect to bony alignment, we measured the lateral distal femoral angle (LDFA) on each plain radiograph [14]. This angle measures the intersection between the anatomic axis and the horizontal line tangential to the subchondral surface of the femoral condyles. The normal value of the LDFA falls between 79° and 83° [15]. We modified the criteria originally suggested by Schatzker and Lambert [3] and modified by Mize [5]. We defined varus deformity as an LDFA 5° above the upper limit of normal (81° ± 2°) [15]. That is, an LDFA > 88° was defined as varus deformity. On the contrary, valgus deformity was defined as an LDFA < 69°, which is 10° below the lower limit of normal. Andriacchi and colleagues [16] and Zhao and colleagues [17] found that loads on the medial compartment of the knee are greater than those on the lateral compartment of the knee during the stance phase of gait. Therefore, we hypothesize that the knee joint is more vulnerable to varus deformity than valgus deformity, due to more loads and stresses on the medial aspect of the knee. Thus, varus deformity should be defined more strictly. Therefore, we evaluated functional outcome with the criteria suggested by Mize, but with a modified definition of varus deformity.

For each fracture, we assessed clinical and functional outcomes of the knee according to 2 scoring systems: Knee Society score (Table 1) [18] and the criteria suggested by Schatzker and Lambert [3], which was further modified by Mize (Table 2) [5]. The Knee Society score included both knee and function scores [18]. The knee score included 4 parameters: pain, range of motion, stability, and deductions. The function score was comprised of functional evaluation of walking and stair climbing. We further modified the criteria suggested by Mize for outcome evaluation [5]. A satisfactory result included an excellent or good score.

**Table 1:** The knee society-based knee and function scores

Parameter	Points	Parameter	Points
Pain		Functions	
None	50	Walking	
Mild or occasional	45	Unlimited	50
Stairs only	40	>10 blocks	40
Walking and stairs	30	5–10 blocks	30
Moderate		<5 blocks	20
Occasional	20	Housebound	10
Continual	10	Inability to walk	0
Severe	0	Stairs	
Range of motion		Normal up and down	50
5° = 1 point	25	Normal up; down holding rail	40
Stability		Up and down holding rail	
Anteroposterior		Up holding rail; inability to walk down	30
<5 mm	10	Inability to climb stairs	15
5–10 mm	5	Subtotal	_
10 mm	0	Deductions (minus)	
Mediolateral		Cane	5
<5°	15	2 canes	10
6°–9°	10	Crutches or walker	20
10°–14°	5	Total deductions	_
15°	0	Function score	_
Deductions (minus)			
Flexion contracture			
5°–10°	2		
10°–15°	5		
15°–20°	10		
>20°	15		
Extension lag			
<10°	5		
10°–20°	10		
>20°	15		
Alignment			
5°–10°	0		
0°–4°	3 points per degree		
11°–15°	3 points per degree		
Other	20		
Total deductions			
Knee score			
(if total is a negative number, score is 0)

**Table 2:** Modification of Mize-Modified Criteria (original criteria suggested by Schatzker and Lambert)

Grading	Description
Excellent	All of the following: loss of flexion, <10°; full extension; no varus, valgus, or rotatory deformity; no pain; perfect joint congruency^a^
Good	No more than any 1 of the following: loss of flexion, >20°; loss of extension, >10°; varus deformity, >5°; valgus deformity, >10°; minimum pain
Fair	Any 2 of the criteria listed in the previous category
Failure	Any of the following: flexion, ≤90°; varus deformity, >10°; valgus deformity, >15°; joint incongruency; disabling pain, irrespective of radiographic appearance

^a^Alignment was determined by measuring the anatomic lateral distal femoral angle (normal range = 79°–83°)

Data were analyzed by using the Student’s *t* test, Fisher’s exact test, and Chi-square test, with the SPSS data analysis program (Version 16, Chicago, IL). We set statistical significance at *P* < 0.05.

## Results

**Fig. 1:**
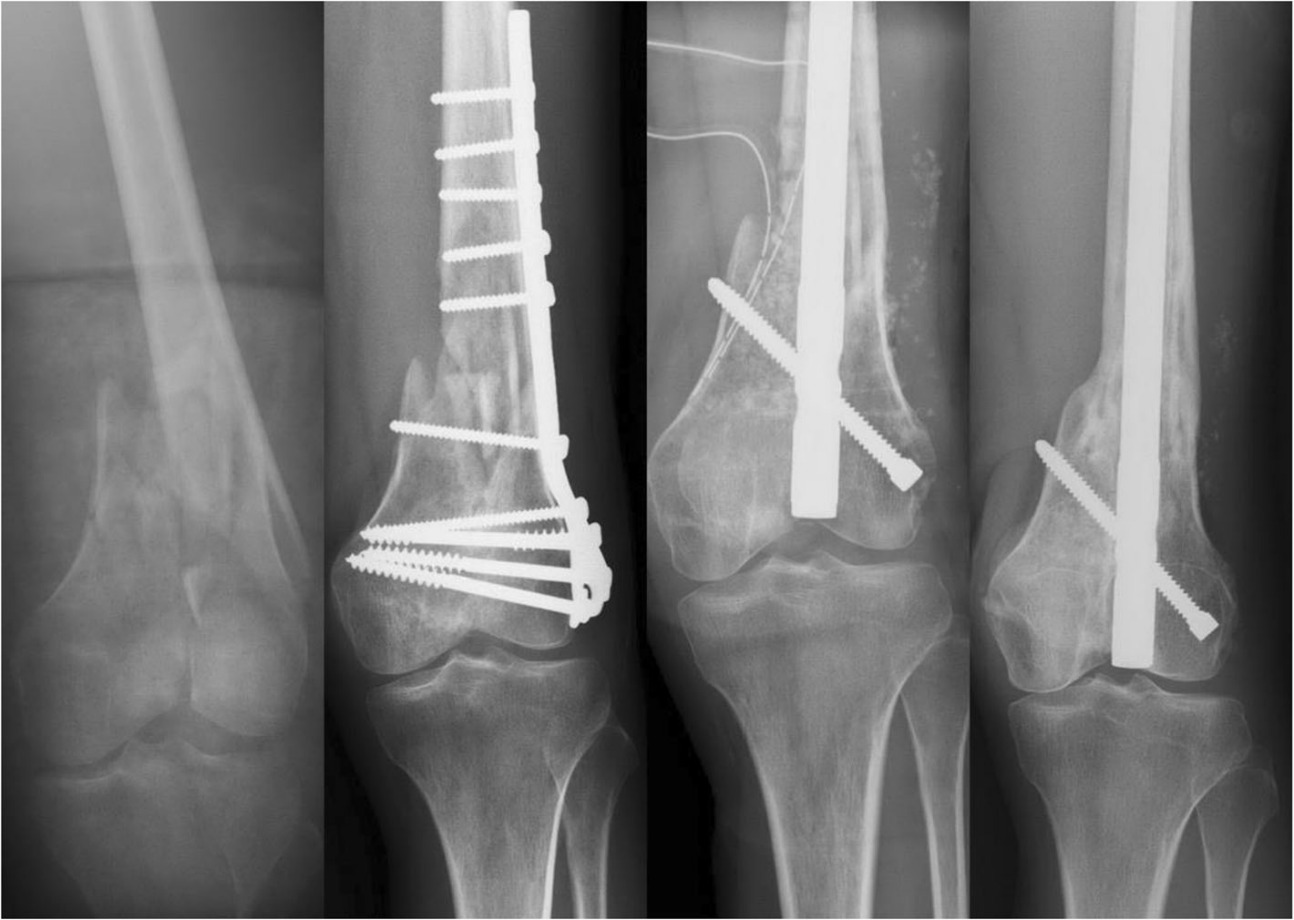
From *left to right*: *left* supraintercondylar fracture; 9-months postoperative; anteroposterior (AP) image taken after revision surgery with intramedullary nail insertion; follow-up AP image, revealing bony union

**Fig. 2:**
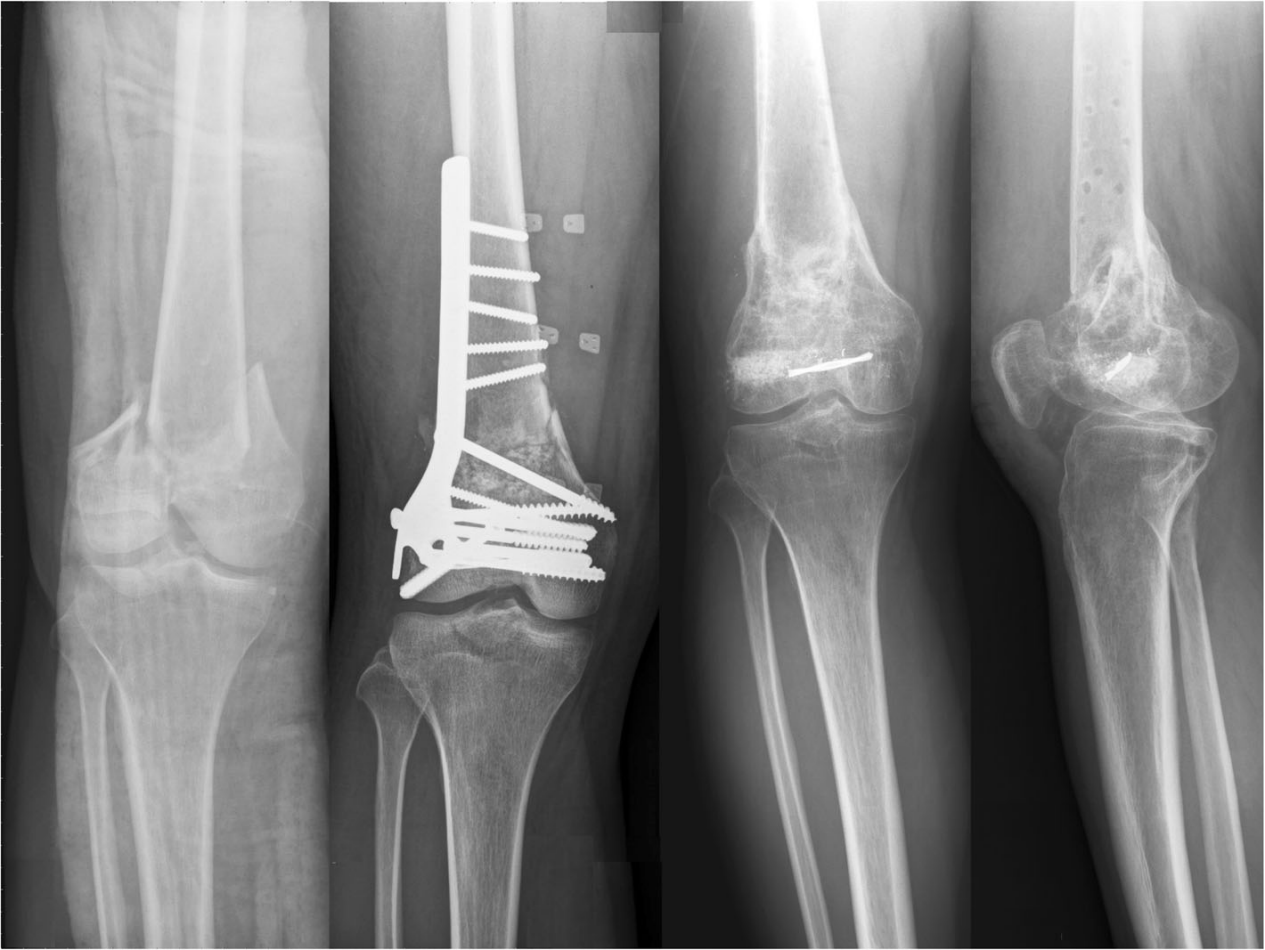
From *left to right*: *right* supraintercondylar fracture; postoperative anteroposterior (AP) image; AP and lateral images taken 1 year after surgery, revealing bony union but varus deformity

In the supraintercondylar group, 30 of 34 patients (17 men and 13 women) received regular OPD follow-up for more than 1 year (range, 13–89 months; average, 29 months). The 1-year follow-up rate was 88.2 %. The average age at the time of fracture was 42.9 years (range, 16–91 years). Fracture occurred on the left side in 10 patients and the right side in 20 patients. As for fracture type, 10 were C1, 14 were C2, and 6 were C3. Twenty-seven patients achieved bony union, resulting in a union rate of 90 % (27/30.) Two patients demonstrated fracture nonunion after postoperative 1-year, and 1 patient who did not show callus formation 9 months after surgery underwent revision surgery with intramedullary nailing (Fig. 1). The average time to union was 6.4 months (range, 2–12 months). Complications included deep infection (2 knees, 6.7 %), knee stiffness (4 knees, 13.3 %), and varus deformity (5 knees, 16.7 %) (Fig. 2). The 2 patients with deep infection were treated with antibiotics and surgical debridement; 1 of the 2 developed chronic osteomyelitis.

**Fig. 3:**
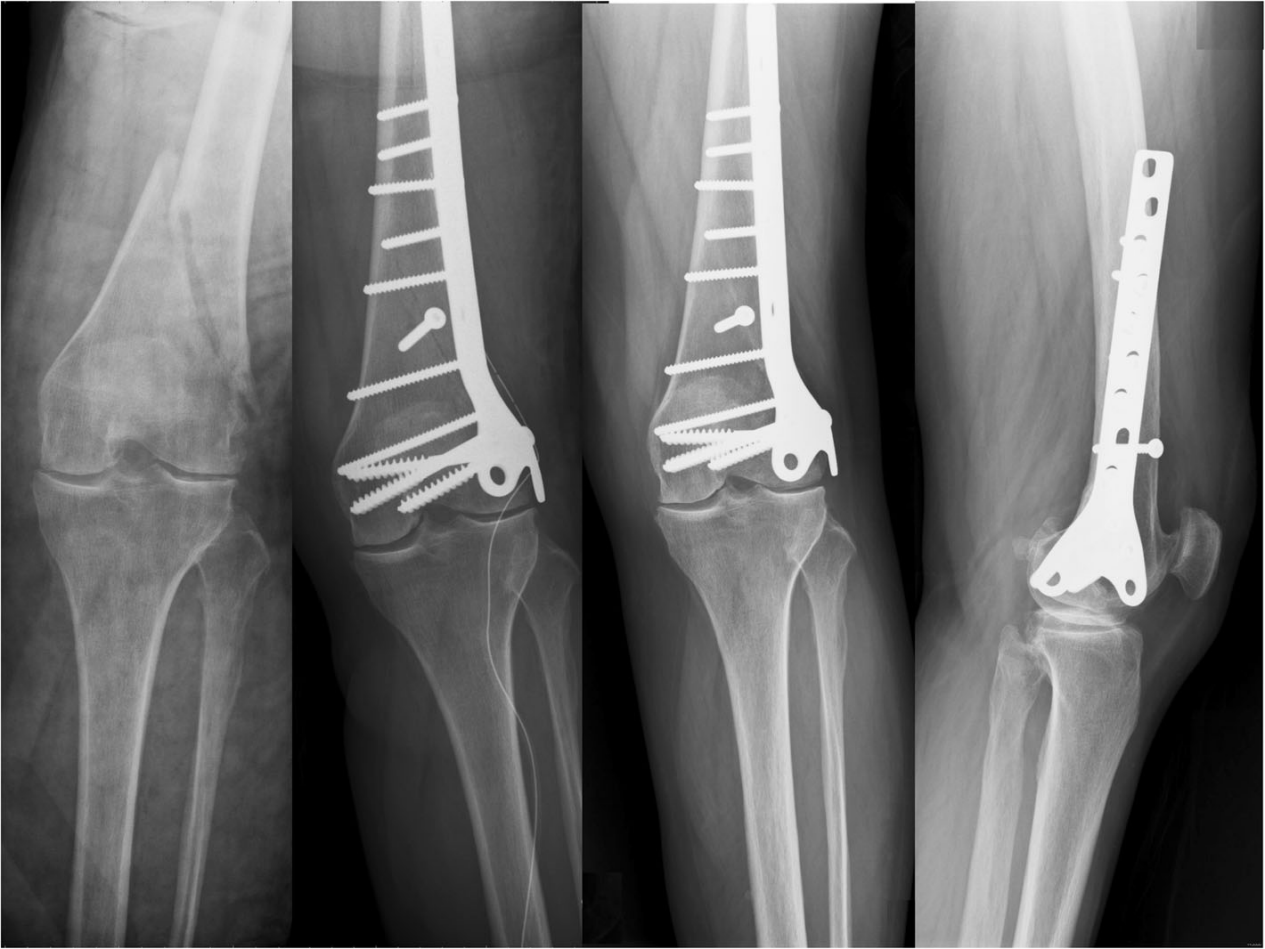
From *left to right*: *left* supracondylar fracture; postoperative anteroposterior (AP) image; AP and lateral images taken 6 months after surgery, revealing bony union and no varus/valgus deformity

In the supracondylar group, 24 of 26 patients (9 men and 15 women) received more than 1 year of OPD follow-up (range, 14–65 months; average, 26 months), with a 1-year follow-up rate of 92.3 %. The average age at the time of fracture was 54.6 years (range, 18–93 years). Fracture occurred on the left side in 11 patients and the right side in 13 patients. As for fracture type, 12 were A1, 12 were A2, and no patient was A3. Twenty-two of 24 patients achieved bony union (Fig. 3), resulting in a union rate of 91.7 %. No patient developed deep infection or varus deformity. However, 2 patients showed nonunion and received revision surgery, eventually achieving bony union uneventfully. The average time to union was 5 months (range, 3–10 months).

In comparing clinical results between supraintercondylar and supracondylar fractures, both groups achieved a high union rate (90 % vs. 91.7 %, *P* = 0.68) and a comparable union time (6.4 months vs. 5 months, *P* = 0.19). However, there were 5 patients in the supraintercondylar group with postoperative varus deformity, but no patient appeared malaligned radiographically in the supracondylar group; this difference was statistically significant (*P* = 0.045).

Functional outcome was evaluated with 2 systems. In the supraintercondylar group, the mean Knee Society knee score was 73.6 (range, 18–100); among them, 50 % (15/30) had an excellent score, 23.3 % (7/30) had a good score, 13.3 % (4/30) had a fair score, and 13.3 % (4/30) had a poor score. The satisfactory rate was 73 % (22/30). The average Knee Society function score was 62.5 (range, 0–100); among these, 46.7 % (14/30) had an excellent score, 3.3 % (1/30) had a good score, 6.7 % (2/30) had a fair score, and 43.3 % (13/30) had a poor score. The satisfactory rate was 50 % (15/30). Evaluation based on the modified Mize criteria revealed 10 % (3/30) of the patients had an excellent score, 40 % (12/30) had a good score, 26.7 % (8/30) had a fair score, and 23.3 % (7/30) demonstrated failure. The satisfactory rate was 50 % (15/30).

In the supracondylar group, the mean Knee Society knee score was 85.5 (range, 42–97); among them, 70.8 % (17/24) had an excellent score, 16.7 % (4/24) had a good score, 8.3 % (2/24) had a fair score, and 4.2 % (1/24) had a poor score. The satisfactory rate was 87.5 % (21/24, *P* = 0.009). The average Knee Society function score was 83.1 (range, 55–100); among these, 50 % (12/24) had an excellent score, 25 % (6/24) had a good score; 20.8 % (5/24) had a fair score, and 4.2 % (1/24) had a poor score. The satisfactory rate was 75 % (18/24, *P* = 0.023). Evaluation based on the modified Mize criteria revealed 29.1 % (7/24) of the patients had an excellent score, 50 % (12/24) had a good score, 16.7 % (4/24) had a fair score, and 4.2 % (1/24) demonstrated failure. The satisfactory rate was 79.1 % (19/24, *P* = 0.09).

The comparisons of clinical and functional outcomes are summarized in Tables 3 and 4.

**Table 3:** Clinical outcomes

Parameter	Supraintercondylar	Supracondylar	*P* value
Union rate	90 % (27/30)	91.7 % (22/24)	0.68
Time to union, mean (range), months	6.4 (2–12)	5 (3–10)	0.19
Complications			
Infection	6.7 % (2/30)	0 %	0.30
Stiffness (knee flexion < 90°)	13.3 % (4/30)	0 %	0.09
Varus deformity	16.7 % (5/30)	0 %	0.045

**Table 4:** Functional outcomes

Parameter	Supraintercondylar	Supracondylar	*P* value
Knee Society Score (Knee score)			
Average score	73.6	85.5	0.009
Excellent	50 % (15/30)	70.8 % (17/24)	
Good	23.3 % (7/30)	16.7 % (4/24)	
Fair	13.3 % (4/30)	8.3 % (2/24)	
Poor	13.3 % (4/30)	4.2 % (1/24)	
(Function score)			
Average score	62.5	83.1	0.023
Excellent	46.7 % (14/30)	50 % (12/24)	
Good	3.3 % (1/30)	25 % (6/24)	
Fair	6.7 % (2/30)	20.8 % (5/24)	
Poor	43.3 % (13/30)	4.2 % (1/24)	
Modified Mize Score			0.09
Excellent	10 % (3/30)	29.1 % (7/24)	
Good	40 % (12/30)	50 % (12/24)	
Fair	26.7 % (8/30)	16.7 % (4/24)	
Poor	23.3 % (7/30)	4.2 % (1/24)	

## Discussion

Fractures of the distal femur account for 6 % of all femur fractures and is clinically challenging. Sufficient mechanical stability is required in the treatment of distal femur fractures; thus, patients should receive early rehabilitation to achieve better clinical outcome. At present, fixation options include nailing systems and plating systems. Intramedullary nailing can be performed in retrograde pattern. Advantages of intramedullary nailing include less extensive dissection, decreased blood loss, and decreased operating time [19]. Some studies suggest that retrograde nailing leads to a higher union rate than that achieved with plating [20–22]. Plating techniques have the advantage of achieving anatomic reduction through a direct view of the fracture site. However, the possibility of invasive incision and soft tissue dissection may lead to complications, including nonunion or delayed union. Locking plate with lateral MIPO approach can have less soft tissue dissection and may not need to expose fracture site. With this technique and modern implant, the possibility of delayed union or nonunion can be decreased. Due to patient’s economic status, condylar buttress plate in the treatment of distal femur still plays a role in our country.

In our previous study, we concluded that the use of a CBP in the treatment of supraintercondylar femur fracture can achieve a union rate of 90 % [12]. However, 5 of 30 patients were noted to have varus deformity after surgery, which equals to an incidence of 16.7 %. In other words, a CBP may not provide enough stability for fixation of supraintercondylar fracture. Davidson and colleagues also reported varus collapse of comminuted distal femur fracture after treatment with a CBP [23]. We further collected data on patients with supracondylar fracture who received treatment with a CBP. We found that these patients achieved a union rate of 91 % and no postoperative malalignment, including varus or valgus deformity. In other words, supraintercondylar femur fractures treated with a CBP lead to 16.7 % postoperative varus deformity, but none occurred in supracondylar femur fractures. The reason for this difference may be that loads on the medial compartment of the knee are much greater than those on the lateral aspect of the knee during the stance phase of gait [16, 17]. The screws that are laterally applied in a CBP may not provide sufficient stability for the medial aspect of supraintercondylar fracture fragments, thus resulting in medial side collapse with varus malalignment. Therefore, double plating may be a possible treatment for supraintercondylar femur fracture. Sanders and colleagues reported good-to-fair functional outcome and a 100 % healing rate in 9 patients with unstable comminuted distal femur fracture treated with double plating and bone grafting [24]. However, in order to fix fracture fragments with plates in both lateral and medial aspects of the femur, there may be too much soft tissue dissection periosteally. Theoretically, it introduces a high nonunion rate. Ziran and colleagues reported plating on anterior and lateral sides through an anterior approach in order to minimize stripping of the medial side of the femur [11]. Nevertheless, supracondylar fracture does not require fixation of medial fragments.

Rademakers and colleagues reported on 67 patients with distal femur fracture treated with a fixed-angle condylar plate [25]. Only 1 patient had nonunion at postoperative 1-year. As for functional outcome, good-to-excellent results were achieved in 84 % based on the Neer score, 75 % based on the Hospital for Special Surgery knee score, and 72 % based on the Ahlbäck score. Dar and colleagues reported on 68 distal femur fractures, including AO/OTA types A and C, treated with dynamic condylar screwing or retrograde intramedullary supracondylar nailing [24]. Union rates of 87.5 % and 89 % were achieved in the dynamic condylar screwing and retrograde nailing groups, respectively. There were no differences between the 2 groups in terms of cumulative union rate, range of motion, complications, or overall results. However, the dynamic condylar screwing group required less time to complete the operation, and there was less blood loss in the retrograde nailing group. Petastodis and colleagues reported on 116 distal femur fractures, including supracondylar and intra-articular involvement, treated with ORIF with 1 of the following 3 implants: CBP, fixed-angle condylar blade plate, or dynamic condylar screwing [10]. They concluded that dynamic condylar screw fixation achieved better functional outcome and lower complication rate compared with CBP and fixed-angle condylar blade plate fixation. Ninety-six percent of patients attained good-to-excellent results. Treatment with dynamic condylar screws is less technically demanding and easier to perform, but removes a large amount of the distal bone stock.

Theoretically, distal femur fractures treated with a locking plate may have more sufficient stability. Therefore, better clinical and functional outcomes may be expected, compared with the current plating system. However, such complications as nonunion, delayed union, and implant failure are not infrequent in distal femur fractures treated with locking plates. Nonunion rates of 0–19 %, delayed union rates of 0–15 %, and implant failure rates of 0–20 % were reported by Henderson and colleagues [11]. There is no consensus over which implant is significantly superior over other implants. Every device has its own advantages and disadvantages. Thus, surgeons should understand the different characteristics of each implant and choose the one they are most familiar working with and that which is most appropriate for the patient.

There are several limitations in our study. The retrospective nature and paucity of patient number make significant relevance less optimal. Thirty-three out of 87 patients were excuded from our series due to loss of follow up and results may be affected if they have enough follow-up periods. Besides, surgeries were not performed by the same surgeon and, therefore, technical familiarity might have affected the results. In order to obtain better evidence of the treatment outcomes, larger patient number and longer follow-up time may be necessary.

## Conclusion

CBPs can achieve a high union rate (>90 %) in the treatment of both supraintercondylar and supracondylar femur fractures. However, 16.7 % of patients with supraintercondylar fracture developed postoperative varus deformity. On the contrary, no patient with supracondylar fracture developed this deformity. Moreover, CBP treatment in patients with supracondylar fractures resulted in better functional outcomes than in those with supraintercondylar fractures.

## Abbreviations

CBP: Condylar buttress plate
LDFA: Lateral distal femoral angle
ORIF: Open reduction and internal fixation.
